# Water-Wheat-Wait: Monitoring wild mallards (*Anas platyrhynchos*) for avian influenza and ESBL-*E. coli* using feed-baited water bins, Germany, 2022 to 2023

**DOI:** 10.1016/j.onehlt.2026.101548

**Published:** 2026-08-17

**Authors:** Francesca I. Rondi, Joaquín Neumann-Heise, Timm Harder, Valerie Allendorf, Klaus Depner, Timo Homeier-Bachmann, Lutz Breuer, Sascha Knauf, Sylvia Dreyer, Anja Globig

**Affiliations:** a Scienze Biotecnologiche Veterinarie, Università degli Studi di Milano, Via Festa del Perdono 7, 20122 Milano, Italy; b Institute of International Animal Health/One Health, Friedrich-Loeffler-Institut, Federal Research Institute for Animal Health, Südufer 10, 17493 Greifswald, Insel Riems, Germany; c Institute of Diagnostic Virology, Friedrich-Loeffler-Institut, Federal Research Institute for Animal Health, Südufer 10, 17493 Greifswald, Insel Riems, Germany; d Institute of Epidemiology, Friedrich-Loeffler-Institut, Federal Research Institute for Animal Health, Südufer 10, 17493 Greifswald, Insel Riems, Germany; e Institute for Landscape Ecology and Resources Management (ILR), Research Centre for BioSystems, Land Use and Nutrition (iFZ), Justus Liebig University Giessen, Heinrich-Buff-Ring 26, 35390 Giessen, Germany; f Centre for International Development and Environmental Research (ZEU), Justus Liebig University Giessen, Senckenbergstraße 3, 35392 Giessen, Germany; g Faculty of Veterinary Medicine, Justus Liebig University, Frankfurterstrasse 106, 35393 Giessen, Germany

**Keywords:** Mallards, Non-invasive surveillance, Highly pathogenic avian influenza (HPAI), ESBL-producing *Escherichia coli*

## Abstract

**Background:**

The southern Baltic Sea coast is an important stopover region for wild waterbirds, particularly between late August and April. These birds can contribute to the maintenance and spread of viral and bacterial pathogens, including highly pathogenic avian influenza viruses (HPAIV) and antimicrobial-resistant bacteria such as ESBL-producing *Escherichia (E.) coli*. The continued circulation of HPAI H5 viruses of the gs/Gd lineage has caused unprecedented global outbreaks in poultry and wild birds. While passive surveillance of sick and dead birds remains the main approach for HPAIV detection, complementary active surveillance of apparently healthy birds using simple, non-invasive sampling is needed to better understand virus ecology and evolution.

**Methods:**

From June 2022 to December 2023, we deployed shallow plastic bins with coastal brackish water and wheat grains along the Greifswalder Bodden shore, Baltic Sea, Germany, to attract wild waterbirds. After bird visits, water samples were analyzed for influenza A virus by RT-qPCR. In July 2022, four samples were cultured for ESBL-producing *E. coli*.

**Results:**

Camera monitoring confirmed that mallards (*Anas platyrhynchos*) were the main visitors, feeding and interacting intensively with the water. 222 water samples were analyzed for viral RNA; 13 (5.9%) were avian influenza virus-positive, including HPAI H5 (clade 2.3.4.4b) and low-pathogenic subtypes H3N8 and H11N9. All four samples yielded ESBL-producing *E. coli*, including one isolate of sequence type ST58.

**Conclusion:**

This non-invasive approach is a promising tool for environmental surveillance of viral and bacterial pathogens, with sensitivity expected to improve through enrichment techniques targeting pathogens in water.

## Highlights

• Feed-baited camera monitored water bins enable non-invasive surveillance of wild waterbirds.
• Water sampling detected HPAIV H5 and LPAIV H3N8 and H11N9 without bird capture.
• ESBL-producing *E. coli,* including ST58, recovered from baited water.
• Low-cost approach suitable for longitudinal wildlife pathogen monitoring.

## Graphical abstract

**Figure:**
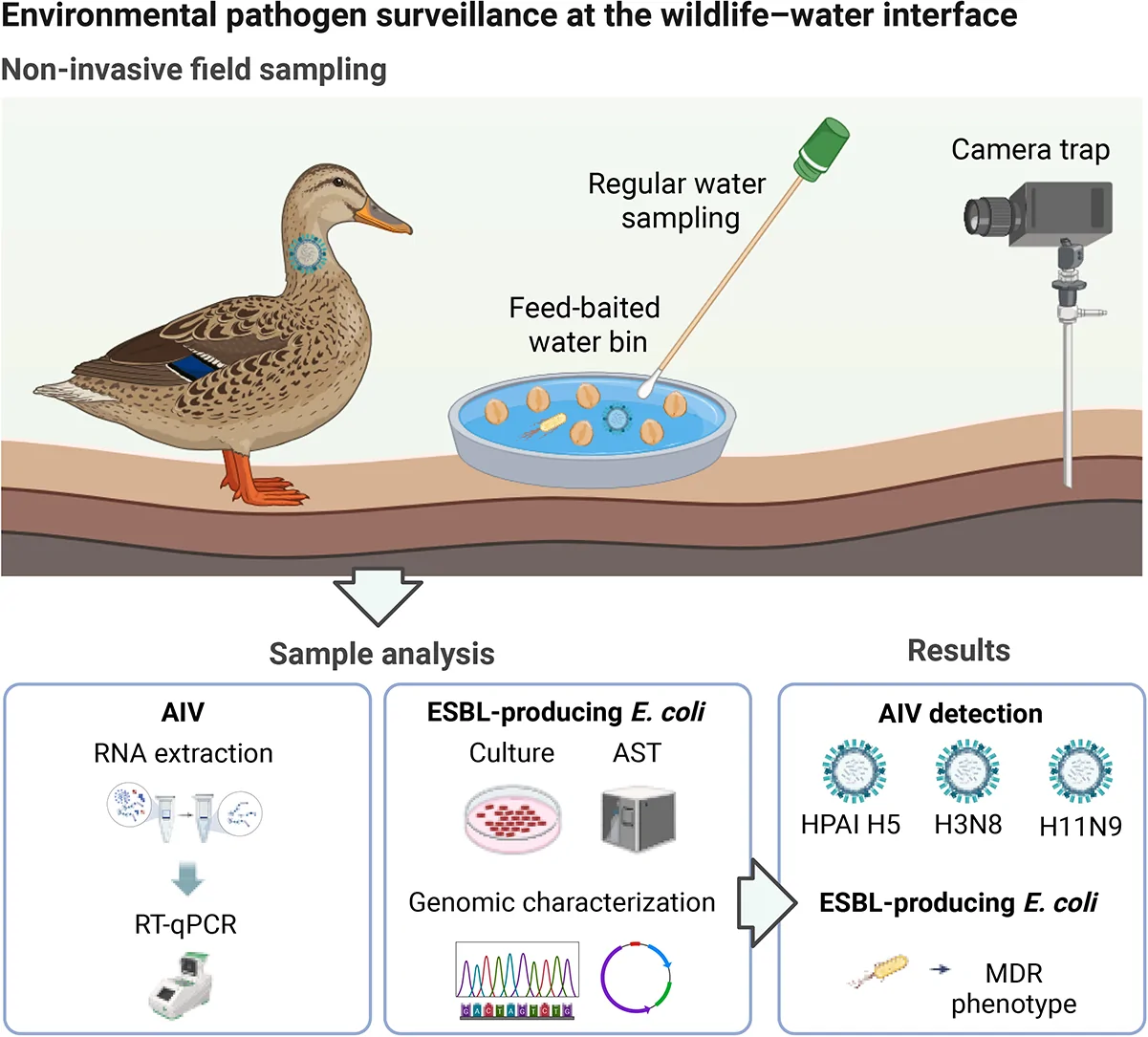

## 1 Introduction

Among wild waterbirds, the orders *Anseriformes* (ducks, geese, and swans) and *Charadriiformes* (gulls, terns and waders) form a natural reservoir system for avian influenza virus (AIV) subtypes H1-H16, H19 and N1-N9 [1]. The highest prevalences are found in dabbling ducks like mallards, wigeons and teals [1]. Transmission of AIV is primarily through the fecal-oral route [2], which makes contaminated feed and water a possible source for infection. In most cases, the infection does not result in clinically severe disease, and many infections may be asymptomatic. Spill-over and subsequent circulation of AIV subtypes H5 and H7 from wild birds to poultry may eventually lead to the emergence of high pathogenicity (HP) phenotype through spontaneous mutation. Such an event is believed to have occurred in 1996 in geese in the Chinese province of Guangdong, giving rise to a HP avian influenza virus (HPAIV) of the H5 subtype [3] (goose/Guangdong-like lineage, gs/Gd). Since late 2003, descendants of this gs/GD lineage have spread globally, initially causing seasonal epizootics and, since 2021, a persistent panzootic in poultry and wild birds [4], [5]. The latest descendants of the gs/GD lineage have caused spillovers into wild and domestic mammals, including dairy cows in the United States of America (USA) [6], [7]. The global distribution, the wide range of species affected and the almost year-round circulation of this HPAIV H5Nx lineage pose increasing threats to the public due to high exposure rates and spillover into humans [8].

Since the emergence and global spread of HPAIV H5 gs/GD lineage in Asia, European Union (EU) Member States are obliged to carry out surveillance programs for HPAIV. Although passive surveillance (interpreted here as examination of sick and dead birds) is still regarded the best tool for HPAIV detection in wild birds [9], additional active surveillance data of apparently healthy live birds of the wild waterbird reservoir is being sought to better understand the implications of wild birds in HPAIV spread and in the epidemiology of AIV in general.

Active surveillance in wild birds is time- and resource-intensive, and it may challenge the conservation of endangered species in specially protected zones. This prompted the need to develop indirect, yet sensitive and informative sampling methods, including the collection of environmental samples such as faeces [10], surface water [10], or feathers [11]. In addition, a sentinel surveillance using captive mallards (*Anas platyrhynchos*) exposed to wild waterbirds in an enclosure situated in their habitat has been evaluated [12]. However, these systems are resource- and labour-intensive, with uncertain impact on sensitivity.

Antimicrobial resistant (AMR) bacteria, which can also be transmitted by migratory birds, are a growing threat to antibiotic treatment of a wide spectrum of bacterial infections [13], [14]. According to the World Health Organization (WHO), bacteria belonging to Extended-Spectrum β-Lactamase (ESBL)-producing *Enterobacterales* are major contributors to the bacterial AMR phenomenon [15]. ESBL enzymes hydrolyse β-lactam antibiotics and render the activity of cephalosporins [16] and penicillins [17] ineffective. Especially third-generation cephalosporin-resistant *Enterobacterales* are tagged “critical” in the WHO Bacterial Priority Pathogens List [18]. Due to anthropogenic habitat degradation and pollution, wild animals become increasingly exposed to antibiotic residues or AMR bacteria [19], compared to settings located more remotely from human settlements [20], [21].

To simplify active surveillance of wild birds for (HP)AIV and ESBL-producing *E. coli*, we developed a new longitudinal, environmental surveillance approach using feed-baited water bins to attract wild dabbling ducks. We hypothesized that water swab sampling from these bins, rather than direct animal sampling, could serve as a proxy for detecting AIV and AMR bacteria. Here, we present data collected over a period of more than one and a half years using this non-invasive approach.

## 2 Methods

### 2.1 Setup and sampling

**Fig. 1:**
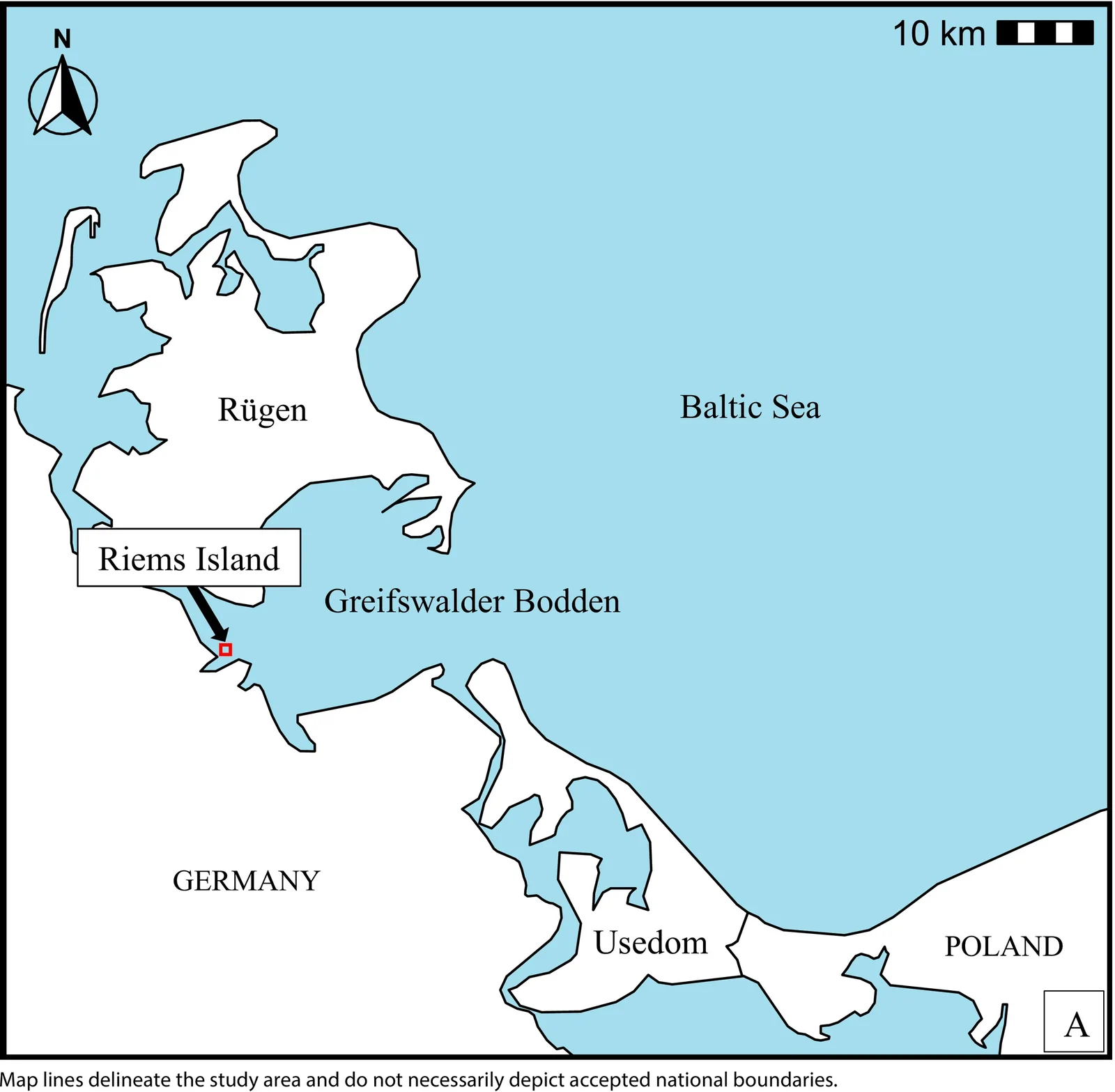

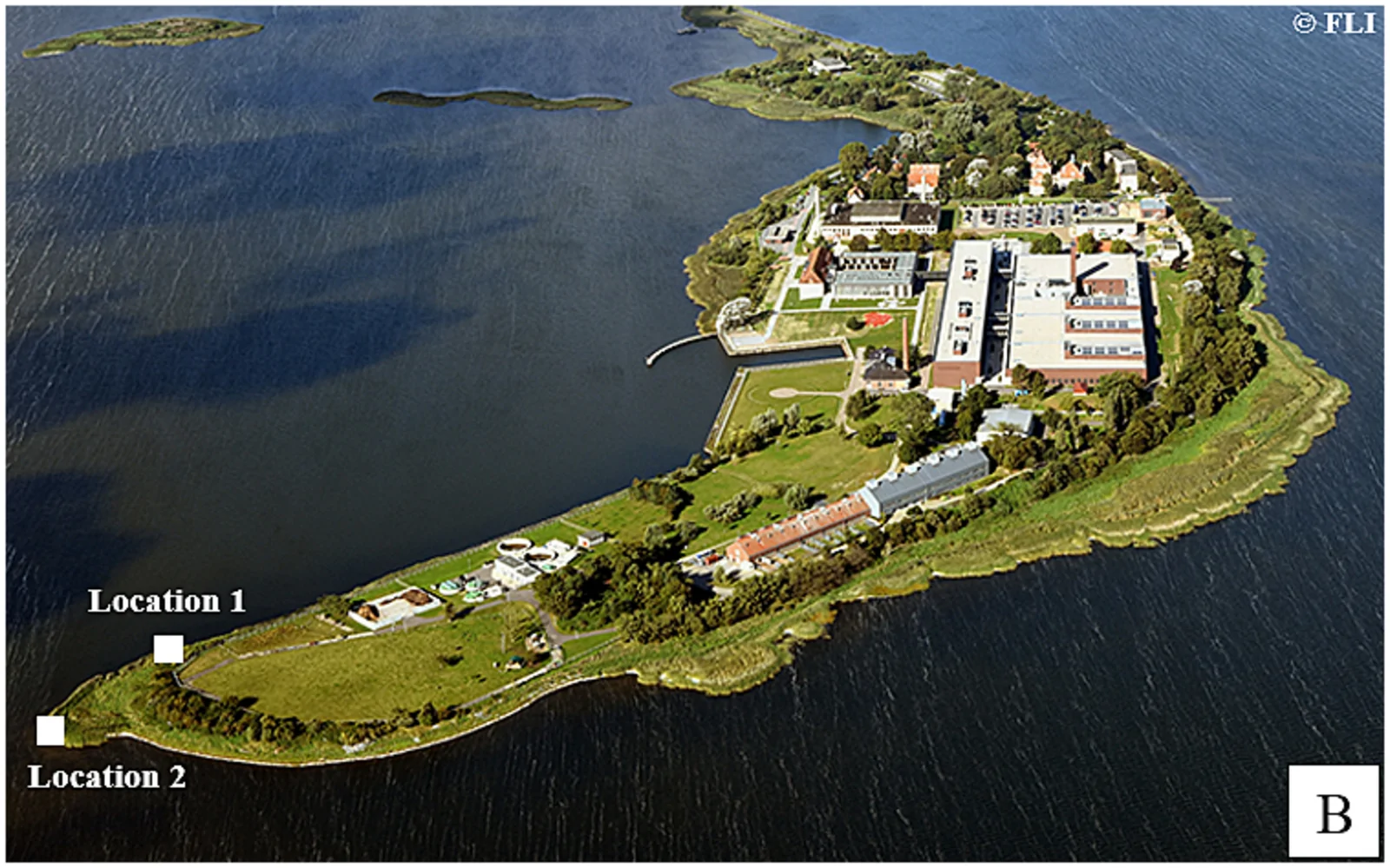
Geographical location of Riems Island and sampling sites. A. The Island of Riems is located at the southern shore of Baltic Sea, in the German Federal State of Mecklenburg Western-Pomerania. The map was created by the authors using R version 4.3.1 (R Core Team, 2023) with the packages ggplot2 v4.0.0 (https://ggplot2.tidyverse.org), sf v1.0–21 (https://r-spatial.github.io/sf), ggspatial v1.1.10 (https://paleolimbot.github.io/ggspatial), and rnaturalearth v1.1.0 (https://docs.ropensci.org/rnaturalearth). B. Aerial view of Riems Island and sampling locations at remote, secluded spots of the island (photo courtesy of Friedrich-Loeffler-Institut, Germany, with permission).

**Fig 2:**
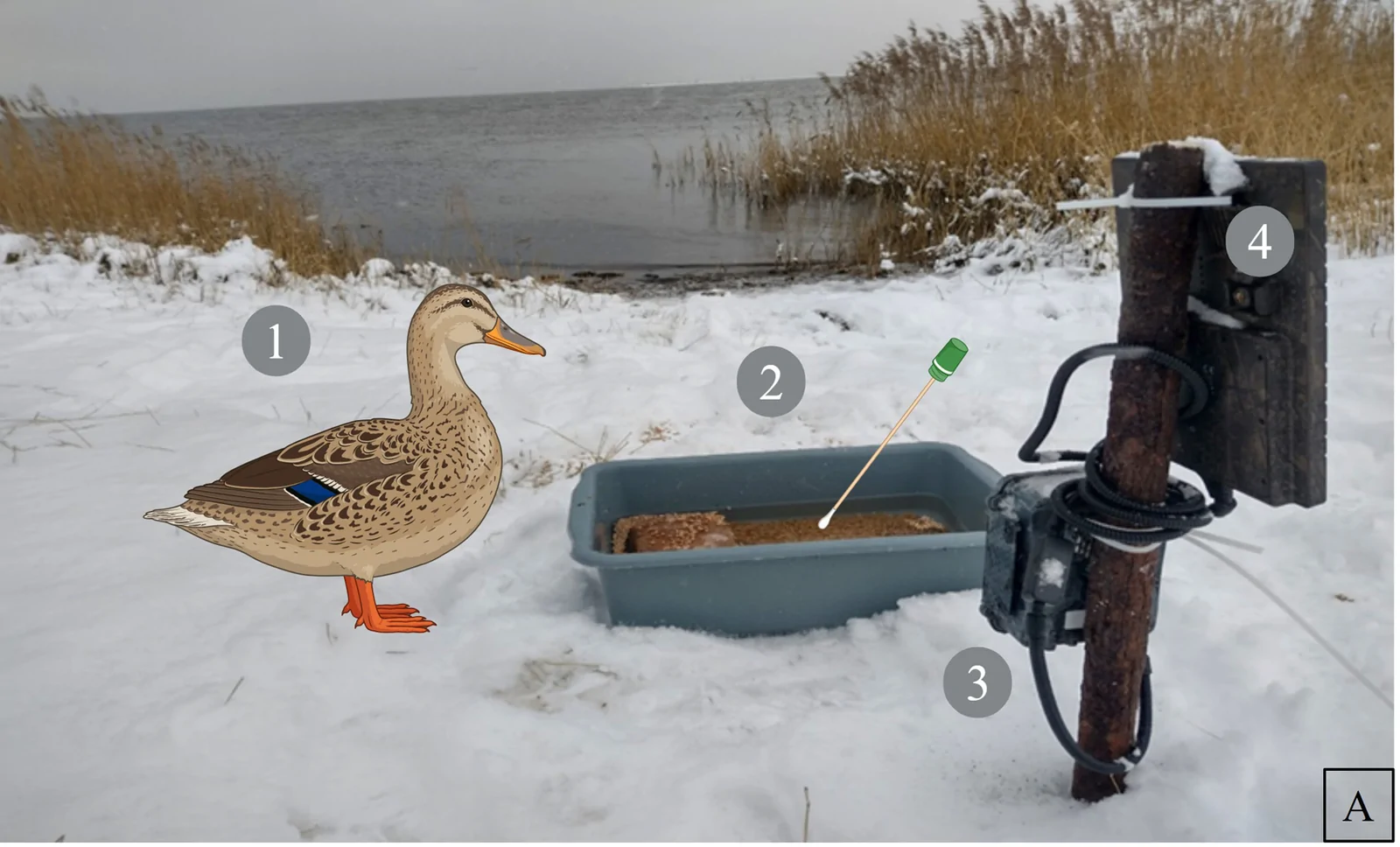

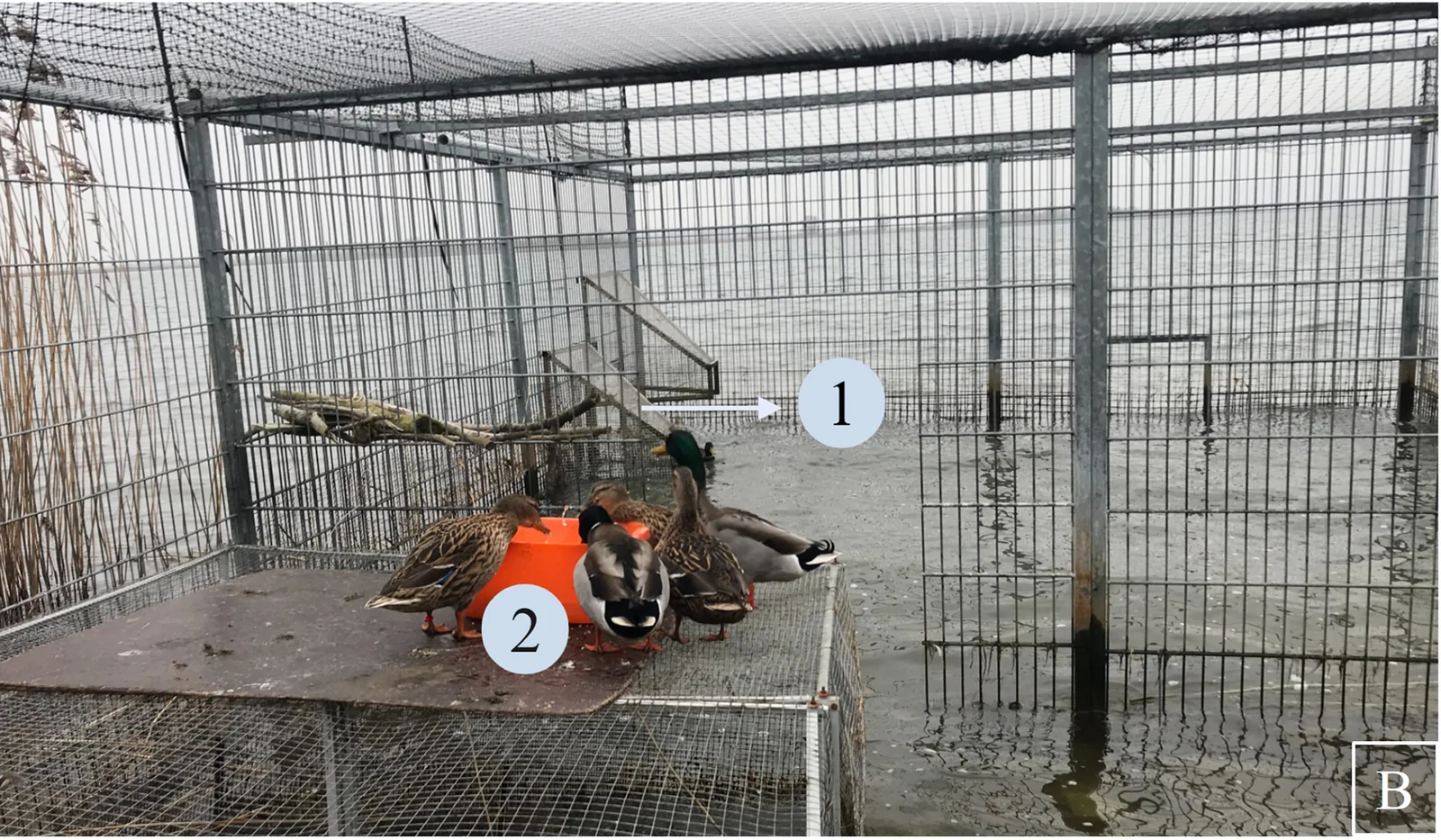
Overview of the field setup of Locations 1 and 2. A. Wild waterbirds in the Greifswalder Bodden were attracted to wheat covered with clear shallow water in bins (1). Swab samples were taken on a general basis two to three times per week (2) from this water for Avian Influenza Virus (AIV) detection and on four occasions for antimicrobial resistant (AMR) analysis. Wildlife encounters were documented using Original Seissiger 12MP Wildcamera® placed at each sampling point (3). The wildlife camera was powered by a solar panel (Original Seissiger Wildcamera®) (4). Using the same methodology as in 2 A, wild waterbirds entered the larger sentinel enclosure through one of its openings (1). The birds then accessed the bin (2) for feeding; the enclosure was used to facilitate access by wild waterbirds while reducing access by wild mammals. Created with BioRender.

Two identical plastic bins, each measuring 48 × 32 × 15 cm, were placed at different locations along the shallow shores of the Island of Riems, southern Baltic Sea, Germany (Fig. 1A, B). The shallow inlets are regionally called “Bodden”. Location 1 (GPS: 54.1806106, 13.3703035) was set inside a sentinel cage (Fig. 2B), a partially enclosed structure that had been used in the past for sentinel surveillance but, at the time of the study, did not house animals and was used only to hold a bin accessible to mallards (*Anas platyrhynchos*). The enclosure was positioned half in shallow water; mallards swam into the enclosure through one of its openings, exited the water, and then swam approximately 3 m across the shallow water of the enclosure to reach the bin (Fig. 2B). Location 2 (GPS: 54.1807343, 13.370910) was set in an open area approximately 3–5 m from the shoreline (Fig. 2A), and birds also had to exit the water to reach the bin. Unlike Location 1, this open setting made the bin accessible to a wider range of wild waterbird species and other free-ranging animals.

Each bin was filled with 5 l of brackish water from the Bodden and 400 g of wheat grains. The bins were properly washed and replenished two to three times per week, except between March and September 2023, when sampling occurred once or twice per week. After filling or replenishing, the bins were left outdoors for one or more days, depending on the season, to allow wild waterbirds to visit and interact with the water. A brick placed on the inside of the bin was used to provide additional stability to the bin and to prevent small rodents, potentially attracted by the wheat, from drowning in the bin. Interactions between the waterbirds and the bins were monitored with wildlife cameras (Original Seissiger 12MP Wildcamera®) powered by solar panels (Original Seissiger Wildcamera®) installed next to each bin (Fig. 2A). These cameras, equipped with motion sensors, automatically captured images both day and night (using a black flash at night). Each week, the memory cards from cameras at both locations were removed, and the data were transferred to a computer for assessment and documentation of relevant contacts between the bins and waterbirds.

Starting from June 2022, water from the bins at Locations 1 and 2 was sampled using ∑-VIROCULT® swabs (Check Diagnostics GmbH, Bad Oldesloe) for AIV analysis. For sampling, the swab tip was immersed and gently moved throughout the water in each bin. In July 2022, the water bin at Location 2 was additionally sampled in four occasions using Sigma transwab® (Check Diagnostics GmbH, Bad Oldesloe) for ESBL-producing *E. coli* investigation, with the swab handled as described above. Personal protective equipment, including face masks, gloves, and rubber boots, was used during all sampling procedures.

### 2.2 Mallard visit counts based on wildlife camera monitoring

All images containing mallards were manually reviewed. For each month, the number of mallards in each image was recorded, and the maximum count per image was noted for further analysis. These records included both direct contact between mallards and the water in the bins, as well as indirect contact. Animals without direct contact were defined as those observed near the bins, showing proximity or exploratory behavior around them, but without any physical interaction with the water or the wheat contained inside. This approach was primarily designed to capture information on mallard visits during their migration and wintering period on Riems Island (typically between September and April each year), while also accounting for visits from the local resident population present year-round.

### 2.3 Environmental data and statistical analysis

Daily temperature data for the city of Greifswald, Germany, from June 2022 to December 2023 were obtained from the Meteostat platform (https://meteostat.net/). The measuring station is located at 54.10° N, 13.40° E, about 6.5 km away from the sampling locations on Riems Island. Statistical analysis was conducted using R version 4.3.1 (R Core Team, 2023), employing the packages dplyr v1.1.4 (https://dplyr.tidyverse.org), lubridate v1.9.4 (https://lubridate.tidyverse.org), and readxl v1.4.5 (https://readxl.tidyverse.org) for data manipulation and handling. Monthly temperature (°C) variation was assessed by first testing the normality of average daily temperature values within each month using the Shapiro–Wilk test. For normally distributed months, mean and standard deviation were reported, and for non-normal months, median and interquartile range were used. Daily average temperature values were aggregated to the monthly level to examine potential relationships with monthly mallard visitation records. The normality of both monthly temperature and visitation data was assessed using the Shapiro–Wilk test. Since monthly temperature data did not meet the assumption of normality, Spearman's rank correlation was applied to investigate associations between both variables.

### 2.4 Virological investigation

The swabs were stored at −80 °C until batches of ten samples were collected for RNA extraction. RNA extraction from individual swabs was performed using the QIAamp RNA Mini-kit (Qiagen, Hilden, Germany). Influenza A virus-generic RT-qPCR targeting the M gene [22], was performed using the AgPath-ID™ One-Step RT-qPCR Reagents kit (ThermoFisher Scientific), as previously described [23]. A heterologous external transcription control was added during extraction [24]. Positive samples were further characterized by RT-qPCR assays targeting fragments of the H5 and N1 genome segments [25]. H5- and N1-negative, but M-positive samples were further subtyped by the RITA assay (Riems Influenza A Typing Array), as detailed elsewhere [23].

### 2.5 Antimicrobial resistance investigation

In line with the recommendation of the WHO's Tricycle Protocol [26], ESBL-producing *E. coli* was used as an indicator for AMR bacteria in this study. Sample investigation was conducted as documented earlier [20]. Briefly, samples were incubated overnight in LB broth (Lennox, Carl Roth GmbH + Co. KG, Karlsruhe) supplemented with 2 μg/ml cefotaxime antibiotic (VWR International GmbH, Darmstadt, Germany) at 37 °C. They were cultivated on CHROMagar Orientation (MAST Diagnostica, Reinfeld, Germany) supplemented with 2 μg/ml cefotaxime, and incubated overnight at 37 °C. Putative ESBL-producing *E. coli* colonies were identified based on colony morphology (pink colour), subcultured for purity and stored at −80 °C in 20% glycerol for further verification and characterization. DNA was isolated using the MasterPure™ DNA Purification Kit for Blood Version II (Lucigen, Middleton, WI, USA) modified for DNA purification from bacteria. Concisely, the procedure started at step 4 of the B2 part of the protocol and with modifications of steps 16 and 17. In step 16, 2 × 100 μl 75% ethanol was used and in step 17 DNA was resuspended in 100 μl TE buffer. Between the steps 16 and 17, the pellet was air-dried to allow evaporation of the remaining ethanol. For suspected ESBL- *E. coli* isolates, Whole Genome Sequencing (WGS) of the isolated DNA was performed at SeqCenter (Pittsburgh, PA, USA) with a sequencing depth of 80× (at least 400 Mbp per sample). Bioinformatic analysis was performed using Multi-Locus Sequence Typing (MLST), ResFinder database (to identify resistance genes) and PlasmidFinder (to identify plasmids). Detailed information is described by Homeier-Bachmann et al. [27] and Dreyer et al. [20].

Antimicrobial susceptibility testing (AST) was performed using the Vitek 2 compact (Biomerieux, Nürtingen, Germany). The susceptibility was tested using software version 9.03.3 and AST-N428 cards. The procedure was performed as described by Dreyer et al. [20].

## 3 Results

### 3.1 Wildlife camera images showed that among waterbirds, only mallards were attracted to feed on the wheat and exhibited distinct seasonal patterns over the study period

**Fig. 3:**
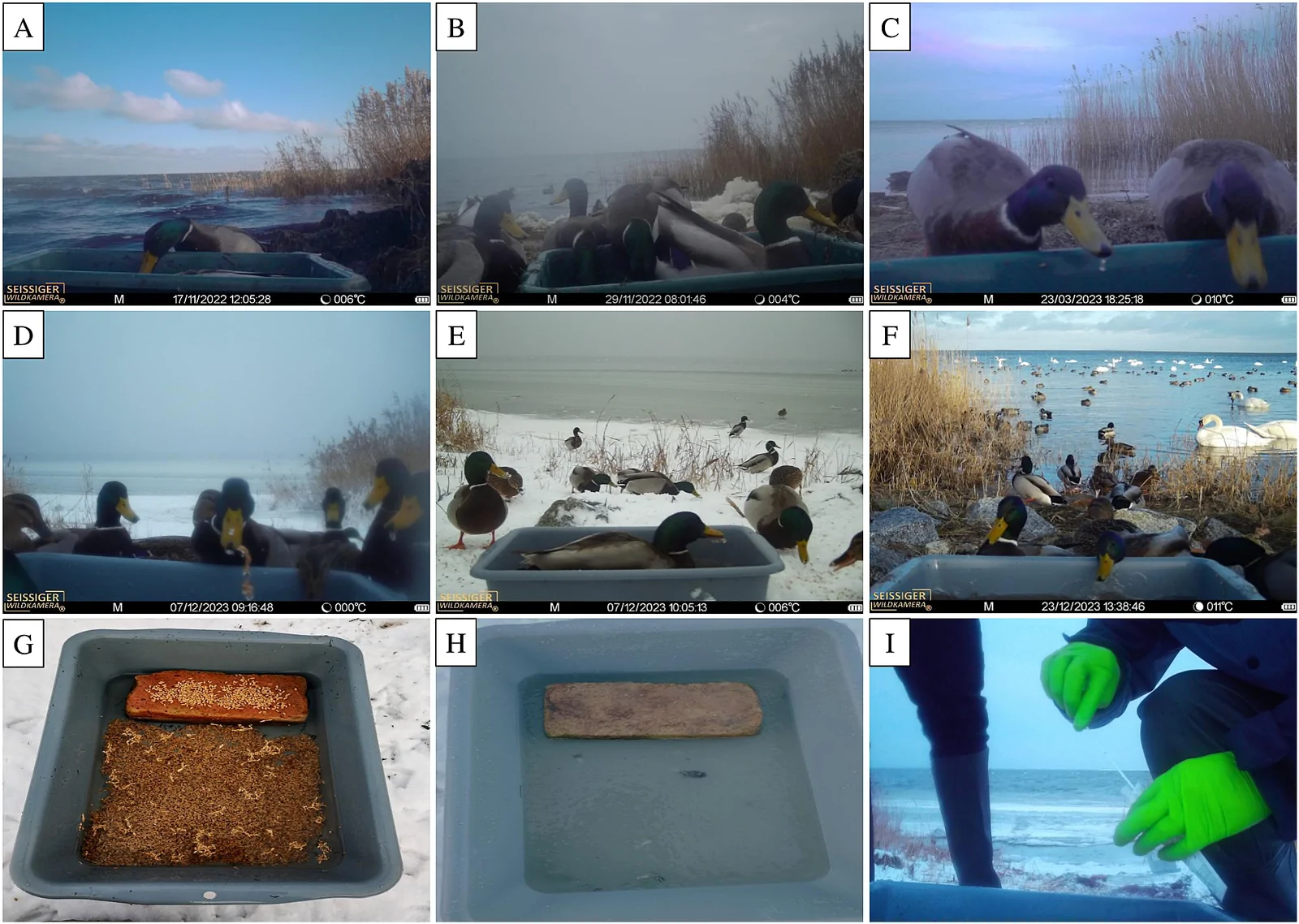
Examples of extensive mallard visits at Location 2, including bin setup before and after visits and swab sampling. Mallards were the only waterbird species seen to be feeding from the bin despite high numbers of other visiting waterbird species (e.g., swans and other duck species), as shown in (F). (G) Illustration of one of the bins with newly prepared water and wheat, (H) and after mallards have fed on the wheat. (I) Collection of a swab sample from the water in the bin after mallards have fed on the wheat.

The feeding setup allowed direct contact between mallards and the water in the bins (Fig. 3A-F). This occurred either through head dipping only (with the rest of the body remaining outside the bins) or through complete entry into the bins, which also included head dipping to feed and could involve excretion (urine/faeces). This facilitated the potential release of viral particles and ESBL-producing *E. coli* from infected or contaminated birds, including via body surfaces.

A total of 129,396 images were collected from wildlife cameras at both locations between June 2022 and December 2023 (see examples in Fig. 3A-F). While at Location 1 only a few mallard ducks (up to 10 individuals captured in a single image per month) were observed visiting regularly throughout the monitoring period, Location 2 showed a higher number and greater diversity of animals approaching the bins. No other duck species than mallards nor swans or gulls were seen feeding on the bins although they were mingling with the mallard ducks in the same surface water close to the bin (Fig. 3F).

**Fig. 4:**
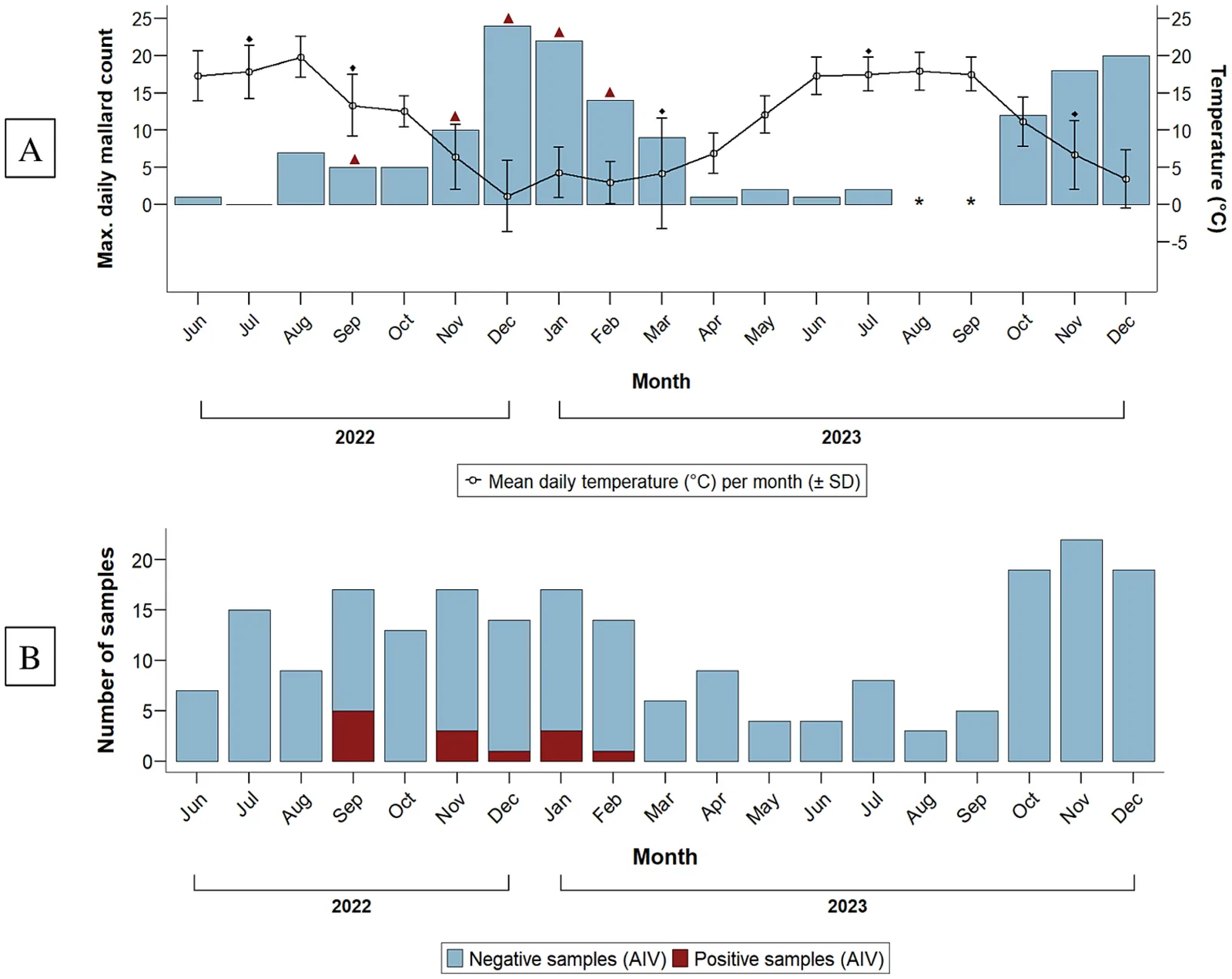
Seasonal patterns of mallard activity and water sample analysis at Location 2 (June 2022 – December 2023). A. Seasonal variation in maximum daily mallard counts per month and monthly temperature trends at Location 2, based on wildlife camera records. B. Monthly total number of water swab samples analyzed and number of samples positive for Avian Influenza Virus (AIV) RNA. Temperature variability in Fig. 4A is represented using standard deviation (SD) for most months, reflecting normally distributed daily temperature data, while interquartile range (IQR) was used in months with non-normal daily temperature distributions, indicated by diamond symbols (◆). Asterisks (*) mark months with no data from the wildlife cameras. Triangles (▲) indicate months in which AIV-positive samples were detected.

Mallard visitations showed distinct seasonal patterns over the study period (Fig. 4A). They were first recorded near the bin at Location 2 in June 2022, and although the first direct-contact observations with the water in the bins occurred in September 2022, such contacts became more frequent between November 2022 and December 2023, based on image records obtained (excluding July 2023, when no direct-contact images were captured, and August–September 2023, when no camera data were collected). The highest number of direct contacts occurred between November 2022 and March 2023, and again from October to December 2023, whereas overall visits—including both direct and indirect contacts—between April and July 2023 were infrequent.

A strong and statistically significant negative correlation was observed between the maximum number of mallards recorded per month and the monthly temperature (using either the mean or the median when appropriate; ρ = −0.77, *p* < 0.001; Spearman's rank correlation, *n* = 17, with minimal tied ranks), indicating that mallard visitations tended to decrease during warmer months (Fig. 4A).

In addition to mallard ducks, various other wildlife species interacted with the wheat in the bins at Location 2, including terrestrial birds such as starlings (*Sturnus vulgaris*), hooded crows (*Corvus cornix*), and small passerine birds such as sparrows (*Passer domesticus*), as well as nocturnal mammalian mesopredators, including raccoons (*Procyon lotor*), European badgers (*Meles meles*), and red foxes (*Vulpes vulpes*). In both locations, also two rodent species, the brown rat (*Rattus norvegicus*) and the striped field mouse (*Apodemus agrarius*), were recorded having direct contact with the wheat in the bins. Additionally, other wildlife species were recorded having indirect contact with the bins at both locations (Supplementary Table S2).

### 3.2 Virological investigation revealed three different subtypes of AIV, including HPAIV H5 clade 2.3.4.4b virus in the swab samples taken from the water between September 2022 and February 2023

Between June 2022 and December 2023, a total of 222 water samples were tested by RT-qPCR. Of these, 13 samples (5.9%) tested positive for AIV, with Ct values ranging from 31.3 to 38.0 (Supplementary Table S1). Positive detections occurred between September 2022 and February 2023, with the highest monthly positivity rate observed in September 2022 (29.4%). After February 2023, no positive samples were detected despite continued sampling efforts (Fig. 4B).

Most of the positive samples (*n* = 10; 71.4%) were obtained from Location 1, where, in addition to two brown rat visits, only mallards had documented contact with water. All positive samples were confirmed by the NRL for AI at the FLI. Subtypes H3N8 (n = 1, September 2022), H11N9 (n = 1, December 2022), HPAIV H5N1 (n = 1, January 2023) and HPAIV H5Nx (n = 1, January 2023) were identified. The remaining positive samples (*n* = 9) could not be subtyped due to low viral loads in the samples (Supplementary Table S1). For the H5 subtype, molecular pathogenicity typing revealed HPAI (clade 2.3.4.4b).

### 3.3 All four samples tested for ESBL-producing *E. coli* harbored cefotaxime resistant phenotypes with resistance to ampicillin and cephalosporin antibiotics

A total of four samples were collected from the bin in Location 2. All bacterial colonies tested showed cefotaxime resistant *E. coli* phenotypes (Table 1). No samples were collected from the water bin in Location 1 for bacteriological investigation.

**Table 1:** Results of Vitek 2 analyses of each isolate. Resistance to antibiotics included in AST card N428 (second column from left).

Antibiotic category	Antibiotics tested	Strain collection IDs			
		3658 [4/7/22]	3659 [22/7/22]	3660 [25/7/22]	3661 [27/7/22]
Penicillins	Ampicillin	R	R	R	R
	Ampicillin/Sulbactam	R	R	R	R
	Piperacillin	R	R	R	R
	Piperacillin/Tazobactam	I	I	I	I
Cephalosporins	Cefuroxime	R	R	R	R
	Cefuroxime-Axetil	R	R	R	R
	Cefotaxime	R	R	R	R
	Ceftazidime	R	R	R	R
Carbapenems	Ertapenem	S	S	S	S
	Imipenem	S	S	S	S
	Meropenem	S	S	S	S
Aminoglycosides	Gentamicin	S	S	S	S
Fluoroquinolones	Ciprofloxacin	S	S	S	S
Glycylcyclines	Tigecycline	S	S	S	S
Sulfonamide-trimethoprim-combinations	Trimethoprim/Sulfamethoxazol	R	S	S	S
	Multi-drug resistant	x			

I = intermediate, R = resistant, S = susceptible, x = multi-drug resistant (MDR) phenotype. Sampling dates are indicated in square brackets [day/month/year].

Antimicrobial susceptibility testing (AST) by Vitek 2 confirmed resistance of all four isolates to ampicillin and cephalosporin antibiotics and susceptibility to carbapenems (Table 1). One isolate revealed a multi-drug resistant (MDR) phenotype, as previously defined by Magiorakos et al. [28] as “the isolate is non-susceptible to at least 1 agent in ≥3 antimicrobial categories”.

To characterize the isolates further on molecular level, whole genome sequencing (WGS) was applied. MLST analysis revealed two different sequence types (STs): ST6521 (*n* = 3) and ST58 (n = 1) (Table 2). All isolates carried three to five plasmids, with Col(pHAD28) and IncFIB as the most common (Table 2).

**Table 2:**
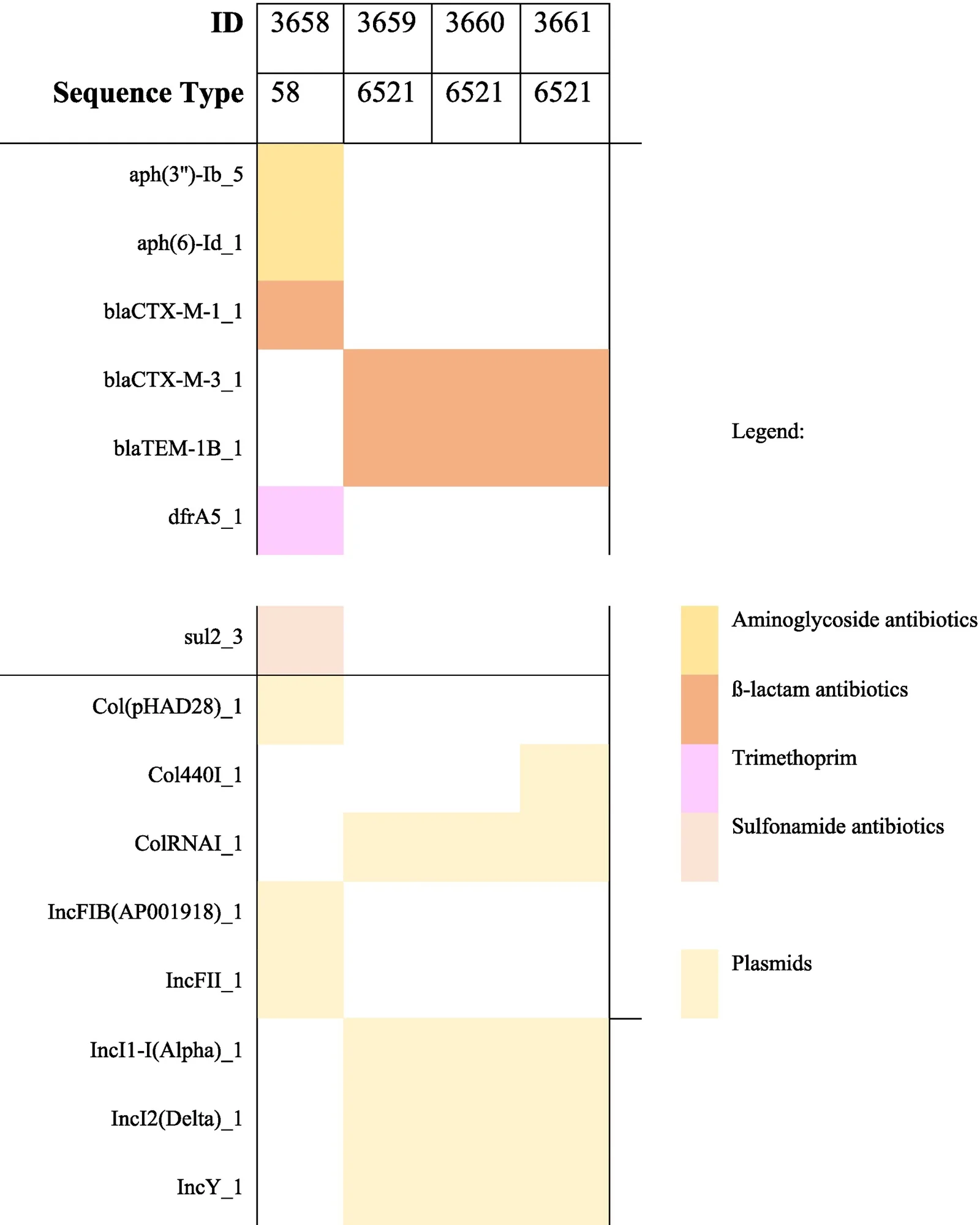
Results of the sequencing analysis for AMR gene (ARG) detection. *E. coli* isolates are indicated by ID number (first line). The second line shows the MLST results of the respective isolate. The ARG section of the table shows the results of the resfinder database. The plasmid genes section of the table shows the results of the plasmidfinder database. Resistance genes and plasmids are presented on the left and colours indicate the antibiotic family or highlighting plasmid results.

## 4 Discussion

This study piloted a practical, active surveillance system for environmental sampling of wild waterbirds using bins containing a small volume of feed-baited water. The sampling targeted avian-derived pathogens and focused on AIV and ESBL-producing *E. coli*.

The approach can potentially be extended to other pathogens excreted by birds, as it facilitates the collection of material likely to contain infectious agents.

It was recently demonstrated that molecular monitoring of bird-populated water bodies can effectively detect widespread antimicrobial resistance genes and occasional influenza virus presence, while also identifying human and avian sources of contamination, using large-volume water samples concentrated via the Skimmed Milk Flocculation (SMF) method [29]. In contrast, the present study shows that small volumes of feed-baited water can also yield pathogen-relevant material, providing a practical and low-effort alternative that achieves detectable results without the need for collecting and processing large water volumes.

Handling these baited bins over a period of 19 months yielded several insights regarding AIV monitoring: (1) Non-invasive, weekly-based water sampling, as opposed to fortnightly sentinel sampling, allowed frequent investigations without disturbing wildlife or compromising animal welfare. (2) Reducing the number of samples proved to be more cost- and labour-efficient than individual animal sampling, although information at the level of individual birds was lost. (3) The use of standard flocked swabs allowed the detection of target pathogens. (4) Finally, multiple AIV subtypes could be detected in subsequent samples, although low viral loads still posed challenges for accurate subtyping, virus isolation, and further characterization, including sequence analyses.

With regard to the second exemplary pathogen, ESBL-producing *E. coli*, the following noteworthy aspects can be highlighted: (1) Water can be considered a carrier, as demonstrated by Lübcke et al. [30], who detected ESBL-producing *E. coli* in 3.3% (1/30) of water samples at the sentinel site. Interestingly, the only ESBL-producing *E. coli* sample they identified was from July 2022 and exhibited the highest biofilm potential, as shown by in vitro assays and the analysis of virulence-associated genes, compared to isolates from other sites. (2) Regarding public health relevance, the present study revealed two different multi-locus sequence types (STs) through WGS, which differed from the STs found in the aforementioned study. While ST 6521 is relatively unknown, ST 58 is considered an emerging high-risk clonal lineage. Some plasmids are well-known carriers of resistance genes, whose characterization is important for public health, although they display weak biofilm-forming capacity [30].

Concerning the challenges encountered while managing the baited bin system during the study period, the following notable aspects can be noted: (1) Sampling complications arose when bins were overturned by raccoons (*Procyon lotor*) and European badgers (*Meles meles*) during the summer, while snow, freezing conditions, and high-water levels disrupted winter sampling. Rain or seawater intrusion may also have affected sample quality. (2) Some logistical and hygiene-related challenges were encountered, as access to drinking water was not possible at these remote sites, making locally available brackish water from the Bodden the only option, which could have introduced contamination from the water itself or from human or environmental sources. Insufficient bin disinfection between samplings may also have facilitated the transfer of ESBL *E. coli* through biofilm formation. (3) Species-specific information was lost, as water sampling does not indicate which species shed the virus [21]. (4) Finally, AIV detection was largely confined to Location 1, suggesting potential site-specific factors influencing pathogen presence.

There are a number of further possible improvements to be addressed in future work to increase efficiency:

• Use clean drinking water whenever feasible to eliminate even minimal risks of environmental contamination or external viral presence. Alternatively, blanks (e.g., brackish water from the Bodden) can be used as controls by collecting water samples from the bins immediately after filling them, that is, before the mallards arrive.
• Apply automated camera image analysis to objectify and quantify contact rates at bins (e.g., counting individuals, recording exact date and time, and distinguishing overlapping subjects).
• Enhance the attractiveness of the setup to other waterbird species to increase the likelihood of detecting pathogens in the water samples.
• Filtering larger volumes of water collected from the bins could increase the amount of viral material available for detection, while subsequent concentration or enrichment prior to RNA extraction could further improve recovery and detection sensitivity.
• Explore the potential for concurrent sampling of fecal material deposited by wild waterbirds in the vicinity of the feed-baited water bins. Such samples could provide an additional source of viral RNA and microbial DNA for pathogen surveillance, complementing the analysis of water samples and potentially increasing the range of detectable pathogens.
• Include avian-specific internal control targets in RT-PCRs signalling avian contact with bins.
• Implement standardised sampling and hygiene measures to reduce the risk of the bins acting as potential pathogen sources for other animals. For example, instead of leaving the bins outdoors for one or more days after filling or replenishing, replenishment could be done early in the morning, followed by a shorter waiting period of 5–8 h for wild waterbirds to interact with the water. After this period, the remaining water would be discarded and the bins stored until the next sampling. This approach could help avoid the sampling complications mentioned above, although it would only be feasible if logistical conditions allow, such as the remoteness of the sampling site or the availability of resources.

## 5 Conclusions

This proof-of-concept study introduced a non-invasive approach for wild waterbirds using feed-baited water bins, which can be applied to detect two zoonotic pathogens: avian influenza virus (AIV) and ESBL-producing *E. coli*. Due to its easy handling and simple setup, this approach can be applied in low-resource settings as a cost-effective, fast, and animal-friendly method, although it is not sufficient to fully understand the complexity of infections and transmission dynamics. The approach described here could potentially be applied to other pathogens shed by wild waterbirds through saliva, faeces, or contamination of plumage or skin, and may also be adaptable for non-invasive testing of wild mammals, such as wild boar and deer (e.g., for Foot-and-Mouth Disease), although this requires further evaluation.

## CRediT authorship contribution statement

**Francesca I. Rondi:** Writing – original draft, Methodology, Investigation, Data curation, Conceptualization. **Joaquín Neumann-Heise:** Writing – review & editing, Writing – original draft, Visualization, Software, Methodology, Data curation. **Timm Harder:** Writing – review & editing, Writing – original draft, Investigation. **Valerie Allendorf:** Writing – review & editing, Writing – original draft, Investigation. **Klaus Depner:** Investigation, Conceptualization. **Timo Homeier-Bachmann:** Writing – review & editing, Methodology, Investigation, Formal analysis. **Lutz Breuer:** Writing – review & editing. **Sascha Knauf:** Writing – review & editing, Supervision. **Sylvia Dreyer:** Writing – review & editing, Writing – original draft, Visualization, Supervision, Methodology, Investigation, Formal analysis, Data curation. **Anja Globig:** Writing – review & editing, Writing – original draft, Visualization, Supervision, Methodology, Investigation, Data curation, Conceptualization.

## Ethical statement

Field sampling was conducted with authorization from the local veterinary authority (Veterinäramt Kreis Vorpommern-Greifswald). No further ethical approval or individual consent was required, as the study did not involve animal experimentats or direct contact with animals.

## Funding statement

This study was funded by the Friedrich-Loeffler-Institut (FLI), the Federal Research Institute for Animal Health under the German Federal Ministry for Food and Agriculture.

## Declaration of competing interest

The authors declare that they have no known competing financial interests or personal relationships that could have appeared to influence the work reported in this paper.

## Data Availability

Daily temperature data from Meteostat used in this study are publicly available and can be accessed at https://meteostat.net/. No proprietary or restricted datasets were used.

## Appendix A Supplementary data

Supplementary material: Table S1. Characteristics of AIV-positive samples. Table S2. Evidence of wild animal with and without direct contact with wheat in water bins, based on wildlife camera recordings at the two bin locations.
