# Supplementary material for: Water-Wheat-Wait: Monitoring wild mallards (Anas platyrhynchos) for avian influenza and ESBL-E. coli using feed-baited water bins, Germany, 2022 to 2023
Source: One Health. 2026 Aug 17;23:101548. doi: 10.1016/j.onehlt.2026.101548 (PMC13542483; doi:10.1016/j.onehlt.2026.101548)
Supplement: Supplementary file 1 [file mmc1.docx]

**Supplemental material**

**Supplementary Table S1. Characteristics of AIV-positive samples.**

| **Positive AIV samples** | **Date**  **(YY-MM-DD)** | **Location** | **CT (M-gene)** | **Sub- and Pathotype** |
| --- | --- | --- | --- | --- |
| 1 | 2022-09-02 | 2 | 36.13 | Nd |
| 2 | 2022-09-02 | 1 | 32.30 | Nd |
| 3 | 2022-09-05 | 1 | 33.70 | H3N8 |
| 4 | 2022-09-08 | 1 | 31.35 | Nd |
| 5 | 2022-09-28 | 1 | 34.19 | Nd |
| 6 | 2022-11-03 | 1 | 36.60 | Nd |
| 7 | 2022-11-18 | 1 | 35.60 | Nd |
| 8 | 2022-11-25 | 1 | 38.00 | Nd |
| 9 | 2022-12-24 | 1 | 33.50 | H11N9 |
| 10 | 2023-01-01 | 1 | 31.33 | H5N1 HP |
| 11 | 2023-01-06 | 1 | 35.50 | H5Nx HP |
| 12 | 2023-01-26 | 2 | 36.55 | Nd |
| 13 | 2023-02-10 | 2 | 36.85 | Nd |

Nd=Not determined due to low viral load.

**Supplementary Table S2. Evidence of wild animal with and without direct contact with wheat in water bins, based on wildlife camera recordings at the two bin locations.**

|  | **Animals in direct contact** | **Animals without direct contact** |
| --- | --- | --- |
| **June**  **2022** | Sparrow (*Passer domesticus*)  Red fox (*Vulpes vulpes*) | Mallard (*Anas platyrhynchos*)  Mute swan (*Cygnus olor*)  Great cormorant (*Phalacrocorax carbo*)  Hooded crow (*Corvus cornix*)  Sparrow (*Passer domesticus*)  Starling (*Sturnus vulgaris*)  Tufted duck (*Aythya fuligula*)  Common kingfischer (*Alcedo atthis*)  Red fox (*Vulpes vulpes*)  European badger (*Meles meles*)  Red deer (*Cervus elaphus*)  Brown rat (*Rattus norvegicus*) |
| **July**  **2022** | Starling (*Sturnus vulgaris*)  Red fox (*Vulpes vulpes*) | Sparrow (*Passer domesticus*)  Reed warbler (*Acrocephalus scirpaceus*)  Starling (*Sturnus vulgaris*)  Eurasian coot (*Fulica atra*)  Red fox (*Vulpes vulpes*)  European badger (*Meles meles*)  Red deer (*Cervus elaphus*) |
| **August**  **2022** | Starling (*Sturnus vulgaris*)  Sparrow (*Passer domesticus*) | Mallard (*Anas platyrhynchos*)  Starling (*Sturnus vulgaris*)  Sparrow (*Passer domesticus*)  Red fox (*Vulpes vulpes*)  European badger (*Meles meles*)  Red deer (*Cervus elaphus*) |
| **September**  **2022** | Mallard (*Anas platyrhynchos*)  Starling (*Sturnus vulgaris*)  Hooded crow (*Corvus cornix*)  Red fox (*Vulpes vulpes*) | Mallard (*Anas platyrhynchos*)  Sparrow (*Passer domesticus*)  Starling (*Sturnus vulgaris*)  Grey heron (*Ardea cinerea*)  Hooded crow (*Corvus cornix*)  Mute swan (*Cygnus olor*)  Red fox (*Vulpes vulpes*)  Red deer (*Cervus elaphus*)  Eurasian otter (*Lutra lutra*)  Brown rat (*Rattus norvegicus*) |
| **October**  **2022** | No encounters recorded | Mallard (*Anas platyrhynchos*)  Sparrow (*Passer domesticus*)  Starling (*Sturnus vulgaris*)  Common kingfischer (*Alcedo atthis*)  Red fox (*Vulpes vulpes*) |
| **November**  **2022** | Mallard (*Anas platyrhynchos*)  Red fox (*Vulpes vulpes*)  Striped field mouse (*Apodemus agrarius*) | Mallard (*Anas platyrhynchos*)  Greater white-fronted goose (*Anser albifrons*)  Grey heron (*Ardea cinerea*)  Mute Swan (*Cygnus olor*)  Common blackbird (*Turdus merula*)  Red fox (*Vulpes vulpes*)  Red deer (*Cervus elaphus*)  European badger (*Meles meles*)  Eurasian otter (*Lutra lutra*)  Striped field mouse (*Apodemus agrarius*) |
| **December**  **2022** | Mallard (*Anas platyrhynchos*)  Hooded crow (*Corvus cornix*) | Mallard (*Anas platyrhynchos*)  Hooded crow (*Corvus cornix*)  Grey heron (*Ardea cinerea*)  Great white heron (*Ardea alba*)  European herring gull (*Larus argentatus*)  Sparrow (*Passer domesticus*)  Mute swan (*Cygnus olor*)  Whooper swan (*Cygnus cygnus*)  Smew (*Mergellus albellus*)  Greater scaup (*Aythya marila*)  Red fox (*Vulpes vulpes*)  Red deer (*Cervus elaphus*)  European badger (*Meles meles*)  Brown rat (*Rattus norvegicus*) |
| **January**  **2023** | Mallard (*Anas platyrhynchos*)  Hooded crow (*Corvus cornix*) | Mallard (*Anas platyrhynchos*)  Hooded crow (*Corvus cornix*)  Grey heron (*Ardea cinerea*)  Mute swan (*Cygnus olor*)  Whooper swan (*Cygnus cygnus*)  Eurasian coot (*Fulica atra*)  Great cormorant (*Phalacrocorax carbo*)  Red fox (*Vulpes vulpes*)  European badger (*Meles meles*)  Eurasian otter (*Lutra lutra*)  Raccoon (*Procyon lotor*) |
| **February**  **2023** | Mallard (*Anas platyrhynchos*)  Hooded crow (*Corvus cornix*)  Raccoon (*Procyon lotor*) | Mallard (*Anas platyrhynchos*)  Hooded crow (*Corvus cornix*)  Great white heron (*Ardea alba*)  Tufted duck (*Aythya fuligula*)  Red fox (*Vulpes vulpes*)  Red deer (*Cervus elaphus*)  European badger (*Meles meles*)  Raccoon (*Procyon lotor*) |
| **March**  **2023** | Mallard (*Anas platyrhynchos*)  Hooded crow (*Corvus cornix*)  Raccoon (*Procyon lotor*)  Brown rat (*Rattus norvegicus*) | Mallard (*Anas platyrhynchos*)  Hooded crow (*Corvus cornix*)  Grey heron (*Ardea cinerea*)  Mute Swan (*Cygnus olor*)  Black-headed gull (*Larus ridibundus*)  Common blackbird (*Turdus merula*)  Red fox (*Vulpes vulpes*)  Red deer (*Cervus elaphus*)  European badger (*Meles meles*)  Eurasian otter (*Lutra lutra*)  Raccoon (*Procyon lotor*) |
| **April**  **2023** | Mallard (*Anas platyrhynchos*)  Hooded crow (*Corvus cornix*)  Red fox (*Vulpes vulpes*)  Raccoon (*Procyon lotor*) | Mallard (*Anas platyrhynchos*)  Hooded crow (*Corvus cornix*)  European herring gull (*Larus argentatus*)  Great cormorant (*Phalacrocorax carbo*)  Common wood pigeon (*Columba palumbus*)  Red fox (*Vulpes vulpes*)  Red deer (*Cervus elaphus*)  European badger (*Meles meles*)  Raccoon (*Procyon lotor*) |
| **May**  **2023** | Mallard (*Anas platyrhynchos*)  Hooded crow (*Corvus cornix*)  Great tit (*Parus major*)  Red fox (*Vulpes vulpes*)  Raccoon (*Procyon lotor*)  European badger (*Meles meles*) | Mallard (*Anas platyrhynchos*)  Hooded crow (*Corvus cornix*)  Grey heron (*Ardea cinerea*)  Mute swan (*Cygnus olor*)  European herring gull (*Larus argentatus*)  Common wood pigeon (*Columba palumbus*)  Red fox (*Vulpes vulpes*)  Red deer (*Cervus elaphus*)  Raccoon (*Procyon lotor*) |
| **June**  **2023** | Mallard (*Anas platyrhynchos*)  Hooded crow (*Corvus cornix*)  Sparrow (*Passer domesticus*)  Common wood pigeon (*Columba palumbus*)  Red fox (*Vulpes vulpes*)  European badger (*Meles meles*) | Mallard (*Anas platyrhynchos*)  Hooded crow (*Corvus cornix*)  Grey heron (*Ardea cinerea*)  Mute swan (*Cygnus olor*)  European Herring Gull (*Larus argentatus*)  Great cormorant (*Phalacrocorax carbo*)  Starling (*Sturnus vulgaris*)  Sparrow (*Passer domesticus*)  Common linnet (*Carduelis cannabina*)  European greenfinch (*Carduelis chloris*)  Red fox (*Vulpes vulpes*)  Red deer (*Cervus elaphus*)  European badger (*Meles meles*)  Raccoon (*Procyon lotor*) |
| **July**  **2023** | Hooded crow (*Corvus cornix*)  Sparrow (*Passer domesticus*)  Red fox (*Vulpes vulpes*)  Raccoon (*Procyon lotor*)  European badger (*Meles meles*)  Brown rat (*Rattus norvegicus*) | Mallard (*Anas platyrhynchos*)  Hooded crow (*Corvus cornix*)  Mute swan (*Cygnus olor*)  Great cormorant (*Phalacrocorax carbo*)  Black-headed gull (*Larus ridibundus*)  Starling (*Sturnus vulgaris*)  Sparrow (*Passer domesticus*)  Red fox (*Vulpes vulpes*)  Red deer (*Cervus elaphus*)  European badger (*Meles meles*) |
| **August**  **2023** | No data | No data |
| **September**  **2023** | No data | No data |
| **October**  **2023** | Mallard (*Anas platyrhynchos*)  Sparrow (*Passer domesticus*)  Raccoon (*Procyon lotor*)  European badger (*Meles meles*)  Brown rat (*Rattus norvegicus*) | Mallard (*Anas platyrhynchos*)  Sparrow (*Passer domesticus*)  Grey heron (*Ardea cinerea*)  Great white heron (*Casmerodius albus*)  Mute swan (*Cygnus olor*)  Great cormorant (*Phalacrocorax carbo*)  Common blackbird (*Turdus merula*)  Song thrush (*Turdus philomelos*) |
| **November**  **2023** | Mallard (*Anas platyrhynchos*)  Sparrow (*Passer domesticus*)  Red fox (*Vulpes vulpes*)  European badger (*Meles meles*)  Brown rat (*Rattus norvegicus*) | Mallard (*Anas platyrhynchos*)  Sparrow (*Passer domesticus*)  Grey heron (*Ardea cinerea*)  Mute swan (*Cygnus olor*)  Common Merganser (*Mergus merganser*)  Red fox (*Vulpes vulpes*)  European badger (*Meles meles*)  Eurasian otter (*Lutra lutra*)  Brown rat (*Rattus norvegicus*) |
| **December**  **2023** | Mallard (*Anas platyrhynchos*)  Red fox (*Vulpes vulpes*)  European badger (*Meles meles*)  Brown rat (*Rattus norvegicus*) | Mallard (*Anas platyrhynchos*)  Mute swan (*Cygnus olor*)  Grey heron (*Ardea cinerea*)  Whooper swan (*Cygnus cygnus*)  Eurasian coot (*Fulica atra*)  Eurasian wigeon (*Anas penelope*)  White-tailed eagle (*Haliaeetus albicilla*)  Red fox (*Vulpes vulpes*)  European badger (*Meles meles*)  Brown rat (*Rattus norvegicus*)  Striped field mouse (*Apodemus agrarius*)  Red fox (*Vulpes vulpes*)  Eurasian otter (*Lutra lutra*) |

Direct contact: animals touched or ate the wheat; no direct contact: animals observed near the bins without physically interacting with water or wheat.
